# Cellular and molecular mechanisms of cell damage and cell death in ischemia–reperfusion injury in organ transplantation

**DOI:** 10.1007/s11033-024-09261-7

**Published:** 2024-03-29

**Authors:** George J. Dugbartey

**Affiliations:** 1 Department of Pharmacology and Toxicology, School of Pharmacy, College of Health Sciences, University of Ghana, Legon, Accra, Ghana; 2 Department of Physiology & Pharmacology, Accra College of Medicine, East Legon, Accra, Ghana

**Keywords:** Ischemia–reperfusion injury (IRI), Organ transplantation, Apoptosis, Necrosis, Necroptosis, Autophagy, Ferroptosis, Pyroptosis, Parthanoptosis

## Abstract

Ischemia–reperfusion injury (IRI) is a critical pathological condition in which cell death plays a major contributory role, and negatively impacts post-transplant outcomes. At the cellular level, hypoxia due to ischemia disturbs cellular metabolism and decreases cellular bioenergetics through dysfunction of mitochondrial electron transport chain, causing a switch from cellular respiration to anaerobic metabolism, and subsequent cascades of events that lead to increased intracellular concentrations of Na^+^, H^+^ and Ca^2+^ and consequently cellular edema. Restoration of blood supply after ischemia provides oxygen to the ischemic tissue in excess of its requirement, resulting in over-production of reactive oxygen species (ROS), which overwhelms the cells’ antioxidant defence system, and thereby causing oxidative damage in addition to activating pro-inflammatory pathways to cause cell death. Moderate ischemia and reperfusion may result in cell dysfunction, which may not lead to cell death due to activation of recovery systems to control ROS production and to ensure cell survival. However, prolonged and severe ischemia and reperfusion induce cell death by apoptosis, mitoptosis, necrosis, necroptosis, autophagy, mitophagy, mitochondrial permeability transition (MPT)-driven necrosis, ferroptosis, pyroptosis, cuproptosis and parthanoptosis. This review discusses cellular and molecular mechanisms of these various forms of cell death in the context of organ transplantation, and their inhibition, which holds clinical promise in the quest to prevent IRI and improve allograft quality and function for a long-term success of organ transplantation.

## Introduction

Ischemia–reperfusion injury (IRI), a critical pathological condition and a major inevitable challenge during organ transplantation, causes significant cell damage and cell death. There are two events of IRI: ischemia, which is the first event, refers to hypoperfusion of tissues or organs due to blockage within arteries. In the field of organ transplantation, ischemia is as a result of donor organ procurement (warm ischemia) and storage in cold preservation solution (cold ischemia) and during engraftment, which causes profound tissue hypoxia, cellular metabolic imbalance and microvascular dysfunction. Therefore, preclinical studies are underway, with the aim of reducing ischemic time to preserve donor organ quality and function. The second event, reperfusion, refers to resumption of blood and oxygen supply to the ischemic tissue. Unfortunately, reperfusion is associated with increased production of reactive oxygen species (ROS; a destructive mediator of cell and tissue injury), excessive inflammatory responses, cell death programs and other pathological processes such as impaired endothelial and mitochondrial dysfunction, calcium overload and autoimmune responses, which altogether, exacerbate ischemic injury [1–3]. Reperfusion injury occurs hours or days after the initial insult. IRI is a major contributor to primary graft dysfunction (PGD), delayed graft function (DGF), slow graft function (SGF), chronic graft dysfunction, graft rejection and other post-transplant complications, leading to increased morbidity and mortality of transplant recipients [4–6]. Despite the efforts made so far in basic and clinical research, detailed mechanisms of cell damage and cell death in IRI have not been fully elucidated. Therefore, further investigations to understand the molecular and other aspects of IRI and its consequences may lead to development of innovative therapeutic strategies as well as improvement in organ transplant procedures. In this review, the author discusses recent mechanistic insights into the various forms of cell death in IRI in organ transplantation, and their inhibition, which holds clinical promise in the quest to prevent IRI and improve allograft quality and function for a long-term success of organ transplantation. The forms of cell death discussed are necrosis, mitochondrial permeability transition (MPT)–driven necrosis, necroptosis, ferroptosis, pyroptosis, parthanoptosis, cuproptosis, apoptosis, mitoptosis, autophagy and mitophagy.

## Cellular mechanisms of ischemia–reperfusion injury

**Fig. 1:**
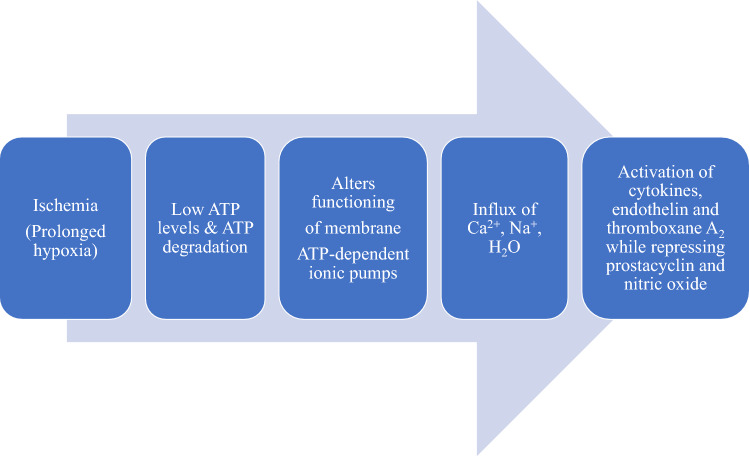
Summary of cellular events during ischemia: Hypoxia due to ischemia disturbs cellular metabolism and decreases cellular bioenergetics through dysfunction of mitochondrial electron transport chain, causing a switch from cellular respiration to anaerobic metabolism and a cascade of events that leads to increased intracellular concentrations of Na^+^, H^+^ and Ca^2+^ and consequently cellular edema

At the cellular level, ischemia causes hypoxia, which disturbs cellular metabolism and decreases cellular bioenergetics through dysfunction of mitochondrial electron transport chain. As illustrated in Fig. 1, depletion in mitochondrial production of adenosine triphosphate (ATP; the source of energy for various cellular functions) during ischemia results in a switch from cellular respiration to anaerobic metabolism and failure of energy-dependent Na^+^/K^+^-ATPase pump on the cell surface as well as detachment of ribosomes. Decreased activity of the Na^+^/K^+^-ATPase pump leads to increased intracellular Na^+^ accumulation, which in turn, inhibits the activity of Na^+^-H^+^. Compensation for the disruption in ionic homeostasis and maintenance of membrane potential results in intracellular Ca^2+^ overload through inhibition of Na^+^-Ca^2+^ exchanger on the cell surface and dysfunction of energy-dependent Ca^2+^-ATPase pump on endoplasmic reticulum, thereby preventing Ca^2+^ re-uptake [7–10]. Hyperosmolarity occurs due to increased intracellular concentrations of Na^+^, H^+^ and Ca^2+^, and consequently leads to water flow into the cytoplasm to maintain osmotic balance, thus resulting in cellular edema [11] (Fig. 1). Intracellular H^+^ accumulation together with increased lactic acid production from anerobic metabolism decreases cellular pH, resulting in metabolic acidosis and impaired enzyme activity and nuclear chromatin clumping. In addition, Ca^2+^ overload causes cell damage via overproduction of ROS, activation of inflammatory cells and cell damage programs as well as increasing cell membrane destruction and mitochondrial dysfunction. Furthermore, ribosomal detachment resulting from ischemia reduces the rate of cellular protein synthesis. The contribution of Ca^2+^ in ischemic injury was confirmed in a rat model of hepatic IRI when Nauta and colleagues [12] observed that intravenous administration of 0.3 mg/kg of verapamil (a calcium channel blocker) prior to induction of hepatic ischemia significantly inhibited Ca^2+^ accumulation in hepatocytes, and prevented mitochondrial Ca^2+^ overload, culminating in preserved mitochondrial respiratory function in another study [13].

**Fig. 2:**
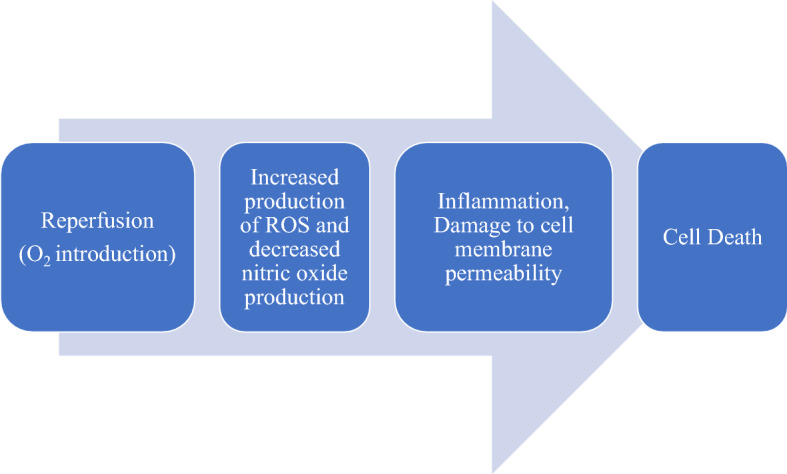
Summary of the pathway leading to cell damage and cell death during reperfusion. Restoration of blood supply to ischemic tissue provides oxygen to the ischemic tissue in excess of its requirement, resulting in over-production of reactive oxygen species (ROS), which overwhelms the cells’ antioxidant defence system, and thereby causing oxidative stress and lipid peroxidation in the cell membranes in addition to activating pro-inflammatory pathways to cause cell death

Re-establishment of blood supply during reperfusion after ischemia provides oxygen to the ischemic tissue more than its requirement, with rapid increase in metabolism. This leads to a series of molecular events that exacerbates the ischemic injury. The re-introduction of molecular oxygen in excess of its requirement increases ROS generation, which overwhelms the cells’ antioxidant defence system, and thereby causing oxidative stress and lipid peroxidation in the cell membranes (Fig. 2). This imbalance between ROS production and the cells’ antioxidant capacity in favor of the former is referred to as oxidative stress. ROS-induced oxidative stress mediates endothelial dysfunction (by destroying vascular endothelial cells), DNA damage and local inflammatory responses through activation of nuclear factor-kappaB (NF-ĸB; an inflammation-related transcription factor) in the vasculature and increased release of pro-inflammatory cytokines, chemokines, adhesion molecules, platelet activating factors, eicosanoid products, and consequently damaging cellular structures, and ultimately resulting in cell death and aggravates the ischemic injury [14].

## Cell death in ischemia–reperfusion injury

It is well-established that cell death is an indisputable indicator of IRI. However, the response of cells to ischemia and reperfusion depends on severity. Moderate ischemia and reperfusion may result in cell dysfunction, which may not lead to cell death due to activation of recovery systems to control ROS production and to ensure cell survival. However, prolonged and severe ischemia and reperfusion induce cell death, which was previously identified to be via two distinct processes: unprogrammed (necrosis) and programmed (apoptosis) [15]. Table 1 is a summary of the various forms of cell death in allograft IRI and emerging protective strategies to mitigate it.

**Table 1:** Types of cell death in allograft ischemia–reperfusion injury and emerging therapeutic strategies

Cell death	Protective strategy	References
Necrosis	Treatment with δV1-1 (PKC inhibitor) Treatment with PP2 (Src PTK inhibitor)	[21, 23, 24]
MPT-dependent regulated necrosis	CypD^−/−^ allograft transplantation Treatment with cyclosporin A and sanglifehrin A	[33, 35–39]
Necroptosis	Treatment with sirolimus Treatment with necrostatin-1 (RIPK1 inhibitor) RIPK3^−/−^ allograft transplantation CypD^−/−^ allograft transplantation	[35, 36, 45, 46]
Ferroptosis	Treatment with lipoxstatin-1 Treatment with ferrostatin-1 Treatment with antioxidants (glutathione, vitamin C & E, alpha-lipoic acid) Treatment with non-toxic iron chelators (e.g. deferoxamine, deferasirox, deferiprone and trimetazidine) Treatment with HO-1/BMMSCs-derived exosomes (HM-exos) Treatment with bone marrow mesenchymal stem cells Pharmacological and genetic inhibition of transferrin receptor 1 (TfR1)	[50, 56, 57, 60–72, 77, 78]
Pyroptosis	Treatment with Ac-YVAD-CMK (caspase-1 inhibitor) Treatment with MCC950 (NLRP3 inflammasome inhibitor) Treatment with disulfiram (gasdermin channel inhibitor)	[89–92]
Parthanoptosis	Treatment with DPQ (PARP-1 inhibitor) Treatment with INO-1001 (PARP-1 inhibitor) Genetic inhibition of PARP-1 Treatment with necrostatin-1	[48, 102, 103]
Cuproptosis	Treatment with dexmedetomidine (ferrodoxin-1 inhibitor) Removal of redox-active copper	[113–115]
Apoptosis/mitoptosis	Mitochondrial transplantation Administration of siRNA directed at p53 Administration of siRNA directed at caspase-3 Administration of shRNA targeted at caspase-8 and Fas Treatment with δV1-1 (PKC inhibitor) Treatment with diannexin Mesenchymal stem cell infusion	[21, 23, 127, 128, 140–142, 145, 146]
Autophagy	Treatment with 3-methyladenine (autophagy inhibitor) Treatment with oligomycin (ATP synthase inhibitor) Treatment with baicalein	[154, 156]
Mitophagy	Upregulation of PINK1, Parkin and BNIP3 genes	[164–166]

### Necrosis as an unprogrammed form of cell death in allograft ischemia–reperfusion injury

Necrosis is an irreversible cell injury and eventual cell death due to several pathological stimuli including hypoxia due to ischemia. Necrosis is the main form of cell death in IRI, especially in reperfusion injury. Necrosis is an uncontrolled cell death, which is associated with swelling of organelles such as mitochondria and endoplasmic reticulum, and plasma membrane rupture resulting in the release of intracellular contents into the extracellular space, as well as activation of inflammasome (cytoplasmic multimeric protein complex) and secretion of pro-inflammatory mediators, leading to eventual cell lysis and tissue damage [16–18]. There is also release of lactate dehydrogenase enzyme after necrosis [19]. Thus, necrosis is associated with loss of membrane integrity and inflammation (necroinflammation). In addition, disruption of lysosomal membrane occurs, leading to the release of proteolytic enzymes into the cell. These enzymes include proteases, DNAases, RNAase, and phosphatases, which degrade DNA, RNA, and proteins, and thereby causing cell death [17]. In the context of organ transplantation, necrotic cell death occurs due to hypoxia during cold ischemia when organ grafts are stored in cold preservation prior to reperfusion. This implies that a longer cold ischemic time increases cell death by necrosis. In fact, every additional hour of cold ischemic time markedly increases the risk of graft failure and mortality of organ transplant recipients [20]. The cellular events leading to hypoxia-induced necrosis begin with decreased ATP production and subsequent events that are presented in the preceding section.

Using a rat model of lung transplantation, for example, 6 h of cold preservation of lung allografts at 4 °C followed by 2 h of reperfusion resulted in the release of pro-inflammatory cytokines and chemokines, with a positive correlation in endoplasmic reticulum stress protein expression and necrotic cell death [21]. As expected, prolonged cold preservation for 18 h caused a further increase in necrosis, with increased upregulated PKC expression. However, treatment with PKC inhibitor (δV1-1) during 18 h of cold ischemic time significantly attenuated lung IRI and improved allograft quality and function by preventing endoplasmic reticulum stress and necrosis when compared to control lung allografts without δV1-1 treatment [21]. This observation corroborates that of a previous study of another model of rat lung transplantation in which cold ischemic time of 6, 12, 18, and 24 h at the same preservation temperature of 4 °C increased graft cell necrosis with increasing cold ischemic time [22]. Microarray analysis after 24 h of cold ischemic time followed by 2 h of reperfusion also showed upregulation in transcripts of transcription factors, adhesion molecules, coagulation factors and pro-inflammatory cytokines that contributed to PGD in a rat model of lung transplantation [23]. This finding was confirmed in a study by Oyaizu and colleagues [24] who reported that 60 min of in situ ischemia and 2 h of reperfusion upregulated the expression of genes related to inflammatory response and increased severity of acute lung injury via activation of Src protein tyrosine kinase (PTK) signaling pathway in a rat model of lung IRI. However, administration of PP2 (Src PTK inhibitor) significantly downregulated the expression of these genes and protected against lung IRI, and thus improved lung function [23].

### Programmed form of necrosis in allograft ischemia–reperfusion injury

While necrosis is traditionally viewed as unprogrammed form of cell death, new evidence has emerged, showing highly regulated form of necrotic cell death in IRI. This includes mitochondrial permeability transition (MPT)–driven necrosis, necroptosis, ferroptosis, pyroptosis, parthanoptosis and cuproptosis [25–29] (Table 1). The molecular mechanisms underlying all these forms of necrotic cell death are discussed in the context of IRI in solid organ transplantation in the next six sections of this review. However, it is important to note that it is not possible to distinguish all these forms of cell death strictly of others, and therefore, a combination of the different forms occur as discussed in this review.

#### MPT-dependent regulated necrosis in allograft ischemia–reperfusion injury

Mitochondrial permeability transition (MPT) pore is a pathological calcium-dependent transmembrane protein complex that is formed in the inner mitochondrial membrane, and serves as major player in mitochondrial swelling and regulated necrosis [30]. MPT-driven regulated necrosis is dependent on cyclophilin D (CypD), a major component and regulator of MPT pore, and triggers the opening of the MPT pore at the intermembrane space, leading to necrosis. Increased mitochondrial matrix levels of ions such as Ca^2+^, and metabolic intermediates as well as ROS promote assembly of MPT pores and further contribute to MPT-mediated regulated necrosis [31, 32]. Using Langendorff technique, results from studies of mitochondria isolated from perfused rat myocardium showed that MPT pores remained closed during 30–40 min of ischemia but opened during 15–60 min of reperfusion, and thus contributed to myocardial injury [33, 34]. In genetic studies with CypD-deficient mice, CypD knockout cardiac allografts showed prolonged recipient survival after transplantation compared to wildtype control grafts [35, 36]. Besides genetic inhibition, pharmacological inhibition of CypD activity with the immunosuppressive agents, cyclosporin A and sanglifehrin A, inhibited MPT pore opening and consequently protected against myocardial IRI and improved cardiac function [33, 37–39]. Considering that cyclosporin A is already in clinical use for maintenance of immunosuppression after organ transplantation, its ability to inhibit CypD and MPT pore opening and improve graft function is a fascinating finding. Taken together, these reports suggest that CypD and MPT pores could be therapeutically targeted to prevent MPT-dependent regulated necrosis and limit transplantation-induced IRI. -

#### Necroptosis in allograft ischemia–reperfusion injury

Burgeoning experimental evidence shows a regulated form of necrosis termed *necroptosis*, shares characteristics of necrosis and some important processes with apoptosis. It occurs due to the activation of receptor-interacting protein 1 (RIP1; a regulator of cell survival and death), mixed lineage kinase domain-like protein (MLKL; a functional substrate of RIP3 kinase) and the assembly of RIP1/RIP3-containing signaling complex (necrosome) that is regulated by the caspase pathway and ubiquitination. Necroptosis is triggered by members of the TNF family including Fas ligand and TNF-related apoptosis-inducing ligand. However, TNF-α is the most studied trigger of necroptosis [40–42]. Just like necrosis, necroptosis also promotes inflammation. Necroptosis-mediated inflammation occurs via several mechanisms including release of cytokines and damage-associated molecular patterns (DAMPs), RIP3- and MLKL-independent activation of inflammasome, and activation of RIP1-mediated cytokine release [43]. Following IRI, DAMPs are released from necrotic cells and bind to pattern recognition receptors (PRR) such as Toll-like receptors, which are predominantly expressed on immune cells, and subsequently activate inflammasomes, and thereby activating immune cells and driving tissue inflammation and further cell death. At the initial stage of necroptosis, interaction between cell death and inflammation creates an auto-inflammation loop that leads to exaggerated cell death and further inflammation. This self-amplifying circuit, referred to as *necroinflammation*, activates systemic inflammation, which leads to graft injury and ultimately graft rejection after transplantation [44]. In a mouse model of heart transplantation, implantation of RIPK3 null cardiac allografts strongly prevented allograft cell infiltrate, inhibited the release of the dangerous DAMP molecule, high mobility group box 1 (HMGB1), and attenuated tissue necrosis after transplantation compared to control group that received wildtype donor allografts. Also, treatment with sirolimus (an immunosuppressant) significantly prolonged RIPK3 null cardiac allograft survival in comparison with control recipient mice [45]. This result supports their in vitro observation, showing that treatment of murine cardiac microvascular endothelial cells with necrostatin-1 (RIPK1 inhibitor) prevented TNF-α-induced necroptotic cell death and release of HMGB1 [45]. Similarly, transplantation of RIPK3 knockout renal allografts prevented inflammatory injury and improved renal function, and thus contributed to prolonged recipient survival. However, in vivo delivery of shRNA directed at caspase-8 in donor mouse kidneys increased necroptosis, HMGB1 release, reduced renal allograft function and accelerated allograft rejection [46]. Also, treatment of proximal tubular epithelial cells with necrostatin-1 inhibited TNF-α-induced necroptosis in vitro [46]. Similar results were reported in vitro using human lung epithelial cells, as well as in remote lung injury after rat kidney transplantation [47, 48]. These findings show that RIPK1/3 contributes to necroptosis of graft cells and allograft injury. Necroptosis was also investigated in graft failure after human liver transplantation. In a clinical trial involving 430 deceased donor liver transplant recipients, a TLR4 single-nucleotide polymorphism, which inhibited binding with HMGB1, reduced the risk of hepatic graft loss after liver transplantation [49]. Mechanistically, pharmacological and genetic inhibition of cyclophilin D, prevented necroptosis of microvascular endothelial cells and inhibited phosphorylation of MLKL downstream [35, 36]. Although necroptosis remains an unexplored pathway in IRI in organ transplantation, its inhibition in donor organs has proven beneficial in experimental organ transplantation, and therefore may provide a similar benefit in clinical organ transplantation.

#### Ferroptosis in allograft ischemia–reperfusion injury

Ferroptosis is a form of regulated necrosis that is iron-dependent and characterized by fatal accumulation of lipid peroxidation, with distinct morphological, biochemical and genetic features compared to other forms of regulated cell death. This form of cell death was identified in cancer cells in 2012 when it was shown that erastin, a small anti-cancer molecule, depleted intracellular glutathione (GSH), inactivated glutathione peroxidase 4 (GPX4; inhibitor of lipid peroxidation), and triggered iron-dependent cell death in a non-apoptotic manner [50–52]. Thus, ferroptosis is based on intracellular iron overload, which causes oxidative stress by enhancing the formation of lipid peroxides, particularly phosphatidylethanolamine-OOH, and increasing ROS production, which overwhelm the cell’s antioxidant capacity, thereby leading to cell damage and cell death [53–56]. For example, it has been reported that iron reacts with hydrogen peroxide during ferroptosis to form hydroxyl-like radicals, hydroxyl and ferric ions, which promote lipid peroxidation, cell injury and cell death, and exacerbate hepatic IRI [57].

Ferroptosis also contributes to inflammation since iron stimulates the release of pro-inflammatory cytokines and induces iron-dependent lipid peroxidation, leading to ROS-induced oxidative damage [56]. Besides lipid peroxidation, free irons promote ferroptosis via non-enzymatic production of ROS. The cytosol and organelles such as lysosomes and mitochondria contain a pool of chelatable and redox-active irons referred to as labile iron pool, which participates in a variety of metabolic processes as well as serving as a catalyst to enhance lipid peroxidation of saturated fatty acids through Fenton reaction to generate ROS [55, 58, 59]. Moreover, mitochondrial production of ROS during stressful conditions involves the role of irons. It should be pointed out that GSH-GPX4 axis is the sole cellular system that preserves cell membrane integrity by repairing oxidized phospholipids, and therefore, GSH depletion or GPX4 inactivation due to intracellular iron overload, leads to loss of cell membrane integrity and ferroptotic cell death. Hence, Friedmann and colleagues [60] demonstrated using GPX4 knockout mice that the absence of GSH-GPX4 axis caused lipid-oxidation-induced acute renal failure and associated death. In the same study, administration of the ferroptosis inhibitor, liproxstatin-1, suppressed ferroptosis in cells, in GPX4 knockout mice, as well as in a mouse model of hepatic IRI [60], indicating that GSH-GPX4 axis is a key antioxidant regulator of ferroptosis. Other inhibitors of ferroptosis such as ferrostatin-1 (Fer-1), and antioxidants (e.g. GSH, vitamin C and E, alpha-lipoic acid) and non-toxic iron chelators (e.g. deferoxamine, deferasirox, deferiprone and trimetazidine) downregulated the expression of prostaglandin-endoperoxide synthase 2 (PTGS2; a ferroptosis marker), which was upregulated in various animal models of IRI, and reduced the levels of intracellular iron, lipid peroxidation, while increasing GSH level, and ultimately attenuating ferroptosis and IRI [50, 56, 57, 61–65].

In a rat model of liver transplantation, a bolus intravenous administration of 50 IU/kg of α-tocopherol emulsion (vitamin E) to donor animal prior to donor liver retrieval and followed by 5.5 h of cold storage and 1 h of reperfusion prevented transaminitis, preserved phagocytic activity of Kupffer cells, sinusoidal endothelial cell linings and intracellular organelles, with fewer surface membrane projections as revealed by electron microscopy compared to control group without α-tocopherol treatment [66]. In addition, α-tocopherol significantly reduced hepatic ROS level, and hepatocyte death by ferroptosis when compared to control group [66]. Using a rat model of liver transplantation with a severe steatotic donor liver and a model of hypoxia-reoxygenation of steatotic hepatocytes, HO-1/BMMSCs-derived exosomes (HM-exos) attenuated IRI in steatotic grafts and inhibited hepatocyte ferroptosis in vitro through increased levels of miR-I24-3p, and downregulated expression of six-transmembrane epithelial antigen prostate 3 (STEAP3; an important enzyme for cellular iron uptake and homeostasis) [67]. Not surprisingly, overexpression of STEAP3 reversed the hepatoprotective effect of miR-124-3p, while HM-exo treatment improved miR-124-3p content in hepatic grafts, which corresponded with decreased STEAP3 expression, and thus reduced ferroptosis. However, HM-exos obtained from cell knocked out for miR-124-3p had no significant effect on ferroptosis [67]. This finding highlights the involvement of ferroptosis in IRI during liver transplantation with severe steatotic donor livers, and suggests a promising approach to treat IRI in steatotic liver grafts by downregulating STEAP3 expression. The result of this study was corroborated by two very recent rat models of steatotic liver transplantation, in which exosomes obtained from heme oxygenase-1-modified bone marrow mesenchymal stem cells inhibited ferroptotic cell death by downregulating the expression of cyclooxygenase-2 (a ferroptosis marker gene), and protected biliary tracts against IRI, resulting in improved steatotic liver function after transplantation [68, 69]. In addition, donor livers treated with bone marrow mesenchymal stem cells during preservation by normothermic machine perfusion showed reduced oxidative damage and ferroptosis of hepatocytes, which was characterized by markedly reduced levels of intracellular ROS and free irons as well as downregulated ferritin expression in a rat model of donation-after-cardiac-death liver transplantation. This resulted in improved graft structure and function compared to untreated control grafts [70]. In a retrospective clinical trial involving 202 pediatric living donor liver transplantation, Yamada et al. [56] also reported high levels of serum ferritin in the donors, which is an indication of iron overload and an independent risk factor for hepatic IRI after liver transplantation. This observation could also implicate ferroptosis in IRI of the liver grafts. In clinical liver transplantation, high expression of E3 ubiquitin-protein ligase HUWE1 in the liver donors was associated with less IRI and improved hepatic allograft function after liver transplantation [71]. This observation was supported by findings from a mouse model of liver IRI in which HUWE1 knockout mice showed exacerbated hepatic IRI with increased ferroptosis [71]. Using an in vitro model, inhibition of HUWE1 in primary hepatocytes significantly increased cellular sensitivity to ferroptosis compared to control hepatocytes. In their mechanistic study, the same authors observed that INO- prevented ferroptotic cell death in HUWE1 knockout mice, and thus mitigated hepatic IRI [71]. This implies that the anti-ferroptotic action of HUWE1 involves targeting TfR1 for ubiquitination and proteasomal degradation, thereby regulating iron metabolism. Therefore, inhibition of TfR1 could provide a platform for the development of novel therapeutic strategies for mitigating IRI following organ transplantation.

Besides the liver, ferroptosis has also been reported in transplantation of other solid organs. In a mouse model of heterotopic heart transplantation, for example, intravenous administration of 10 mg/kg of ferrostatin-1 (Fer-1; ferroptosis inhibitor) to recipient mice 1 h prior to reperfusion significantly ameliorated cardiac allograft IRI by reducing the levels of pro-ferroptotic hydroperoxy-arachidonoyl-phosphatidylethanolamine (a lipid peroxide), preventing the increase in cardiomyocyte cell death and suppressing neutrophil accumulation in the cardiac allograft via inhibition of TLR4/Trif-dependent signaling pathway in coronary vascular endothelial cells after heart transplantation when compared to control group without Fer-1 administration [72]. In a separate experiment by the same authors involving coronary artery ligation-induced myocardial IRI, Fer-1 prevented ferroptotic cell death, markedly reduced myocardial infarct size, improved left ventricular systolic function, and decreased left ventricular remodeling via TLR4/Trif inhibition relative to control mice without Fer-1 treatment [72]. Thus, TLR4/Trif-dependent signaling pathway provides additional insight into the ferroptotic pathway, and could represent a new therapeutic target in mitigating IRI after organ transplantation. Most recently, Zhang and associates [73] conducted a clinical investigation involving 20 patients who suffered chronic renal allograft dysfunction. Following biopsy and nephrectomy of the transplanted kidneys, immunohistochemical staining revealed accumulation of lipid peroxidation, which was evidenced by increased renal expression of 4-HNE (a product of lipid peroxidation) compared to normal kidney tissues. This observation indicates ferroptosis, since this form of cell death is characterized by accumulation of lipid peroxidation products. Ultrastructurally, ferroptosis manifested as mitochondrial atrophy, high mitochondrial membrane density, and reduced mitochondrial ridges in renal tubular epithelial cells of kidneys from the chronic allograft dysfunction group [73]. The authors extended their clinical observation to a mouse model of kidney transplantation and in vitro study using renal tubular epithelial cells. In both experimental models, treatment with TNF-α induced ferroptotic cell death by upregulating the expression of interferon regulatory factor (an inflammation-related transcription factor) in renal tubular epithelial cells, leading to inhibition of GPX4 transcription [73]. This finding was supported by later clinical studies in which analysis of RNA-sequencing data from kidney transplant biopsies showed that ferroptosis-associated genes mediate allograft rejection after kidney transplantation [74–76] as well as in mice [76]. Similarly, cold storage and reperfusion of human lungs activated ferroptosis-related signaling pathway by increasing the contents of iron, lipid peroxidation and cyclooxygenase-2 in the lung tissues, and downregulating the expression of lung GPX4 and other antioxidant proteins, and thereby altering mitochondrial morphology [77]. The finding supports the in vitro observation by the same authors involving human bronchial epithelial cells [77]. Using a mouse model of lung transplantation in which lung allografts were treated with liproxstatin-1 (Lip-1; ferroptosis inhibitor) during 24 h of cold storage followed by 2 h of reperfusion, inhibition of ferroptosis markedly attenuated lung IRI, preserved lung allograft architecture and improved pulmonary function after transplantation in comparison with control lungs without Lip-1 treatment [77]. Thus, Lip-1 could be considered in the future as a new anti-ferroptotic and cytoprotective agent for improved organ preservation against IRI. In an in vitro study involving human pancreatic islets, Bruni and colleagues [78] also demonstrated the effect of ferroptosis on islet viability and function, and possibility of safe human islet transplantation in the future. In their study, treatment of human islets with the ferroptosis-inducing agents, erastin and RSL3, resulted in significant death and reduction in islet function as revealed by lactate dehydrogenase release and stimulation index (a marker of islet function) respectively [78]. However, these effects were prevented following pretreatment with Fer-1 and the iron chelator, desferrioxamine [78]. Taken together, although ferroptosis was recently identified as a form of regulated necrosis, with limited literature, preclinical and clinical studies so far implicate ferroptosis as a significant contributor to cell damage and cell death in IRI during organ transplantation. Therefore, targeting ferroptotic pathways with inhibitors or non-toxic iron chelators in organ transplantation could serve as a potential and novel approach to limit IRI, and improve allograft quality and function after transplantation.

#### Pyroptosis in allograft ischemia–reperfusion injury

Pyroptosis has been recently identified as a new form of regulated necrosis that is inherently inflammatory and induced by various pathological stimuli, including ischemia and reperfusion. The term *“pyroptosis”* originates from the Greek word *“pyro”*, which means *“fire”* (referring to the properties of inflammation), and *“ptosis”* which means *“falling”* (referring to other forms of programmed cell death). Pyroptosis is characterized by loss of plasma membrane integrity and triggered by activation of various inflammasomes that activate caspases, resulting in formation of plasma membrane pores, osmotic swelling and rupture of plasma membrane and consequent release of cytoplasmic contents [79–84]. It was previously thought that pyroptotic cell death involves canonical (caspase-1-dependent) and non-canonical (caspases-4-, 5- and 11-dependent) pathways. However, extensive studies on this form of regulated necrosis have revealed other pathways that are mediated by other caspases such as caspase-3 and 8, as well as granzyme [81–85]. In the canonical pathway of pyroptosis, caspase-1 in inflammasomes is activated and cleaves gasdermin D (GSDMD; a pore-forming protein and executioner of pyroptosis), which forms non-selective pores on plasma membranes. These pores are referred to as *“gasdermin channels”*, and their formation leads to release of interleukin-1β (IL-1β), IL-18, water and eventually cell lysis [81, 86, 87]. Unlike the canonical pathway, cleavage of GSDMD in the non-canonical pathway is mediated by stress-induced activation of caspase-4, 5 and 11 to form gasdermin channels and exacerbate inflammatory response [82]. In caspase-3/8-mediated pathway of pyroptosis, stressful stimuli such as TNF-α or chemotherapy drugs activate caspase-3 and 8 to specifically cleave GSDMD and gasdermin E (GSDME), which promote inflammasome formation in addition to forming gasdermin channels [83, 84]. Granzyme-mediated pyroptosis occurs when GSDMB and GSDME respectively cleaved by granzymes A and B (cytotoxic caspase-like proteases) [85, 88].

A few studies have reported the contribution of pyroptosis in IRI in experimental and clinical organ transplantation. In a rat model of orthotopic lung transplantation, treatment of lung allografts with leukocyte-depleting filter and highly selective caspase-1 inhibitor (Ac-YVAD-CMK) during 4 h of ex vivo perfusion before 1 h of cold storage at 4 °C produced a substantial improvement in lung allograft structure and function including microvasculature, with less inflammation as seen in markedly reduced levels of perfusate pro-inflammatory cytokines such as IL-6 and lung mRNA for IL-6, IL-1β and TNF-α compared to control lung allografts without treatment [89]. In addition, flow cytometric analysis revealed 26% of trapped pyroptotic monocytes in the perfusates, which migrated from the lungs of the treated group [89]. In a clinical study of donation-after-cardiac death lung transplantation by the same authors, trapped pyroptotic cells were also observed in the lung perfusates following the same treatment during ex vivo lung perfusion prior to transplantation, with significantly reduced caspase-1-positive cells in the lung allografts [89]. Using a rat model of orthotopic allogeneic and isogeneic liver transplantation, daily intraperitoneal administration of Ac-YVAD-CMK to recipients of allogeneic liver transplantation significantly reduced rejection activity index to a level comparable to that of isografts, which positively correlated with markedly reduced levels of lung and serum IL-1β and IFN-γ mRNA compared to allografts without Ac-YVAD-CMK treatment [90]. Similarly, intravenous administration of MCC950 (a selective inhibitor of NLRP3 inflammasome) to recipient pigs of allogeneic livers transplantation followed by intravenous infusion of MCC950 in the same recipient pigs on post-transplant days 2 and 3 after 2 h of cold preservation (4-6 °C) of liver grafts by hypothermic machine perfusion resulted in significantly lower levels of serum IL-1β and TNF-α, reduced hypertransaminasemia, improved liver allograft structure and function, and ultimately contributed to prolonged transplant recipient survival compared to untreated control group [91]. Mechanistically, the graft-protecting effect of MCC950 was associated with inhibition of NLRP3/caspase-1/IL-1β, a typical pyroptotic pathway. Considering the significant role of GSDMD in pyroptotic cell death, disulfiram, a medication commonly known for the treatment of chronic alcoholism, has recently been identified as an inhibitor of gasdermin channels following its anti-pyroptotic action, which involved prevention of pro-inflammatory cytokine release in a mouse model of lipopolysaccharide-induced septic death [92]. This promising laboratory finding suggests that disulfiram could be a potential pharmacological tool to provide more insights in the context of pyroptosis and its role in organ transplantation. Although pyroptosis has not been extensively studied in the context of organ transplantation, a growing body of experimental evidence implicates its involvement in various IRI models of transplantable solid organs [93–97]. Collectively, the preclinical and clinical findings suggest that inhibition of pyroptotic pathway could provide effective and innovative therapeutic approach to attenuate IRI and its associated complications after organ transplantation. However, given that the few studies that investigated the role of pyroptosis in organ transplantation have focused on canonical (caspase-1) pathway of pyroptosis, further studies are needed to investigate the other pathways of pyroptotic cell death in IRI in organ transplantation.

#### Parthanoptosis in allograft ischemia–reperfusion injury

The term “parthanoptosis” was derived from “*Thanatos*” in ancient Greek religion and mythology, which personifies death. This form of regulated necrosis is characterized by hyperactivation of the DNA repair enzyme poly(adenosine diphosphate-ribose) polymerase-1 (PARP-1; the first member of the PARP superfamily) by a variety of pathological stimuli such as ROS, alkylating agents, calpain, increased Ca^2+^ concentration, increased mitochondrial permeability and DNA damage [98, 99]. Under physiological conditions, PARP-1 activation contributes to cellular homeostasis by mediating DNA repair, genomic stability and transcription. However, under pathological conditions that cause genomic stress such as ischemia and reperfusion, PARP-1 is hyperactivated and depletes cellular content of nicotinamide adenine dinucleotide (NAD^+^; a critical coenzyme in cellular energy production), and thus impairs cellular metabolism, and consequently ATP production. This causes nuclear release of mitochondria-toxic PAR polymer, and thereby inducing nuclear translocation of active, truncated death effector apoptosis-inducing factor from the mitochondria, leading to chromatin condensation and large-scale DNA fragmentation. Such mitochondrio-nuclear translocation results in parthanoptosis in a caspase-independent and ATP-independent pathway [100, 101].

Studies of parthanoptosis mainly focused on the central nervous system in the context of neuronal damage and neurodegeneration. However, one study reported the contribution of parthanoptosis in experimental organ transplantation. In a rat model of kidney transplantation, storage of renal allografts in preservation solution at 4 °C for 24 h followed transplantation, resulted in remote lung injury, which was characterized by upregulated expression of PARP-1 (parthanoptosis) and RIP1 and 3 (necroptosis) in the lung tissue [48]. However, daily intraperitoneal administration of the necroptosis inhibitor, necrostatin-1, to recipient animals reduced parthanoptosis and necroptosis death by downregulating these markers of cell death [48]. This interesting experimental result could provide the molecular basis for combination therapy that targets parthanoptosis and other forms of cell death in IRI to prevent post-transplant complications. Apart from necrostatin-1, genetic inhibition of PARP-1 and PARP-1 inhibitors such as DPQ, have also been reported to reduce parthanoptosis. Using PARP-1 knockout mice, cold storage of kidneys in preservation solution at 4 °C for 48 h followed by reperfusion prevented parthanoptosis of renal tubular epithelial cells and reduced renal damage compared to wildtype mice [102]. In addition, treatment of donor kidneys with DPQ during 48 h of cold preservation significantly downregulated PARP-1 nuclear expression in tubular epithelial cells of kidneys from wildtype mice, resulting in reduced parthanoptosis, attenuated IRI and improved renal function in comparison with wildtype control mice without DPQ treatment [102]. In a randomized, placebo-controlled, single-blind clinical trial involving 40 patients with ST elevation myocardial infarction undergoing primary percutaneous coronary intervention, bolus intravenous administration of the PARP-1 inhibitor, INO-1001, reduced in vitro PARP-1 activity in the plasma of these patients, with a corresponding decrease in the levels of plasma C-reactive protein and IL-6 [103]. The promising result from these experimental studies and clinical trials could serve as the platform for a novel therapy for IRI in organ transplantation and other clinical conditions of IRI by targeting parthanoptosis.

#### Cuproptosis in allograft ischemia–reperfusion injury

Another form of regulated necrosis that has been identified and currently under investigation in the context of organ IRI is cuproptosis. It is a copper-triggered mode of mitochondrial cell death, in which excess intracellular copper binds to lipoylated enzymes in the mitochondrial tricarboxylic acid cycle, leading to aggregation of fatty acylated proteins (mediated by intracellular ferredoxin-1), reducing iron-sulfur cluster protein levels, mitochondrial dysfunction and proteotoxic stress, and ultimately cell death [104–106]. Thus, cuproptosis is characterized by copper dependence and regulation of mitochondrial respiration. Copper is a trace element, which is essential in hemoglobin synthesis, bone formation and other processes, including development of internal organs as well as in the functions of central nervous system and immune system [107–110]. However, increased intracellular concentration of copper damages vascular endothelial cells by triggering ROS production and accumulation, which disrupts cellular function [111, 112]. Emerging experimental evidence shows that cuproptosis plays a crucial role in the development and progression of many diseases, including organ IRI, which could be applied in the context of organ transplantation. In two rat models of IRI in which isolated rat hearts were subjected to 20 min of warm ischemia followed by 30 min of reperfusion, removal of redox-active copper prior to ischemia prevented post-ischemic cardiac oxidative injury and enhanced recovery of myocardial function, with significantly reduced levels of hydroxyl radicals and efflux of lactic dehydrogenase compared to control hearts that were loaded with copper [113, 114]. Recently, Guo and colleagues [115] also reported that pharmacological inhibition of cuproptosis with dexmedetomidine targeted at ferredoxin-1 markedly reduced copper levels, preserved mitochondrial function, increased antioxidant status and reduced cuproptosis-related proteins in in vitro and in vivo models of cerebral IRI. Although research on the mediatory role of cuproptosis in organ IRI is in its infantile stage, its inhibition is expected to become a potential therapeutic approach for clinical conditions involving IRI, including organ transplantation.

### Programmed form of non-necrotic cell death in allograft ischemia–reperfusion injury

#### Apoptosis and mitoptosis in allograft ischemia–reperfusion injury

Apoptosis is a tightly-regulated ATP-dependent process of programmed cell death that is activated by hypoxia due to ischemia and during ROS generation in reperfusion. Apoptosis is characterized by cell shrinkage, formation of apoptotic bodies, cell membrane blebbing [116, 117]. Ultrastructurally, there is DNA fragmentation, nuclear and cytoplasmic condensation, chromatin rearrangements, myofibril loss and disarray, contour irregularities and amorphous dense bodies, as well as damaged cell–cell-contacts, most of which are due to activation of a Ca^2+^-dependent endonuclease and other enzymes [118, 119]. The initiation and execution of apoptosis are dependent on caspases, a family of proteolytic enzymes. There are two major apoptotic pathways that are mediated by caspases: the extrinsic and intrinsic pathways, which interact with each other to exert their influence. The extrinsic pathway, also known as death receptor pathway, depends on extracellular molecules (death ligands) such as tumor necrosis factor-alpha (TNF-α), Fas ligand, and TNF-related apoptosis inducing ligand (TRAIL), which bind to death receptors (e.g. TNFR1, Fas) in the cell membrane and undergo a conformational change in the death-inducing signaling complex (DISC) to initiate cytotoxic signals by converting procaspases to caspases. Caspase-8, a crucial initiator caspase in the extrinsic pathway, directly cleaves and activates effector (executioner) caspases such as caspase-2, 3, 7 and 9, which induce apoptosis of damaged cells via proteolysis [120–122]. The intrinsic pathway, also called mitochondrial pathway, is activated under stressful conditions such as hypoxia during ischemia, and negatively impacts mitochondrial integrity, resulting in release of pro-apoptotic factors (e.g. cytochrome c, endonuclease G, apoptosis-inducing factor) from the mitochondria into the cytoplasm to induce apoptosis in a caspase-dependent and independent pathway [120–122].

In IRI, for example, there is increased mitochondrial translocation of cytoplasmic pro-apoptotic proteins such as BAD, BAX, BID, and BAK, which induce mitochondrial release of cytochrome c, endonuclease G, apoptosis-inducing factor, and other pro-apoptotic factors into the cytoplasm. The release of these pro-apoptotic factors from the mitochondria promotes their additional release. The mitochondria then become disintegrated due to loss of membrane potential resulting from these pro-apoptotic factors and disruption of the mitochondrial electron transport chain, thereby contributing to apoptosis in a process of programmed destruction termed *mitoptosis* [120–122]. Nuclear translocation of endonuclease G and apoptosis-inducing factor causes DNA fragmentation and caspase-independent apoptosis while cytochrome c binds to apoptotic protease-activating factor 1 to activate caspase cascade via formation of apoptosome and subsequent activation of caspase 9 [123, 124]. Following ROS-induced DNA damage during reperfusion, increased expression of p53 (a tumor suppressor protein) induces the formation of PIDDosome (a protein complex) to execute apoptosis via caspase-2 activation [125–127]. Therefore, inhibition of caspase-2 with small-interfering (si)RNA reduced ROS production, prevented mitoptosis and apoptosis of cardiomyocytes, and thus attenuated myocardial IRI [126].

Considering the role of mitochondria in apoptosis in IRI, mitochondrial transplantation, also known as mitochondrial transfer, is emerging as a novel approach to protect and preserve mitochondrial integrity against IRI and other disease conditions involving mitochondrial dysfunction. Mitochondrial transplantation is a process where exogenous isolated functional mitochondria are taken up by damaged cells to recover dysfunctional mitochondria. In an in vitro model of renal tubular injury, for example, treatment of damaged human proximal tubular cells with mitochondria enhanced proliferative capacity and significantly increased ATP production, along with preserved physiological polarization, and markedly reduced toxicity and ROS production compared to control cells that were treated with placebo [128]. The authors conducted a separate experiment using a non-survival ex vivo model of donation-after-cardiac death kidney transplantation, in which donor kidneys were treated with mitochondria prior to perfusion at room temperature for 24 h. Compared to placebo-treated control kidneys, Raman spectroscopy of perfusate samples revealed fewer molecular species in mitochondria-treated kidneys, which indicates stability. Additionally, mitochondrial transplantation resulted in less kidney damage while analysis of RNA sequencing showed increased mitochondrial bioenergetics and downregulated expression of pro-inflammatory genes [128]. Also, supplementation of organ preservation solution with mitochondria-targeted AP39 and sodium thiosulfate at 21ºC and 4ºC for 4 and 24 h respectively and reperfused for 4 h prevented apoptosis, protected against IRI and improved renal graft function in *ex viv*o porcine and rat models of kidney transplantation [129, 130]. These experimental findings suggest that targeting the mitochondria by mitochondrial transplantation or by pharmacological approach could offer an innovative strategy against IRI in organ transplantation as well as in other aspects of mitochondrial medicine. Although these empirical findings are promising, mitochondrial transplantation therapy is a controversial topic, as there are several unsolved issues including mitochondrial immunogenicity, methodology for using preserved mitochondria, mitochondrial long-term storage, mitochondrial yield and purity, maintenance of mitochondrial integrity during transplantation, and transplant rejection [131]. For example, immune responses in mitochondrial transplantation have become a subject of debate. While no immune response was observed following syngeneic and allogeneic mitochondrial transplantation in murine, lagomorph and porcine models [132–134], several other studies have reported significant immune response following mitochondrial transplantation, which was characterized by activated vascular endothelial cells, markedly elevated plasma levels of pro-inflammatory cytokines, chemokines, mitochondria-derived DAMPs, which correlated with early allograft dysfunction and increased graft rejection [135, 136]. This controversy underscores the fact that mitochondrial transplantation therapy is still far from being fully effective, and that there is the need for further understanding of the mechanisms underlying an immune response and other challenges associated with mitochondrial transplantation.

Using a rat model of syngeneic kidney transplantation, intravenous administration of siRNA directed at p53 (siP53) after 40 min of warm ischemia and 5 h of cold storage (cold ischemia) and prior to reperfusion resulted in significantly less apoptosis of proximal tubular epithelial cells and cast formation compared to control group that received saline [127]. In addition, siP53 preserved renal function, which was evidenced by significantly reduced serum creatinine level and increased renal blood flow relative to control rats [127]. Similarly, intravenous injection of siP53 following 4 h of ischemia in rats markedly reduced the upregulated p53 mRNA and protein expression and attenuated p53-mediated apoptosis in a dose- and time-dependent manner [137]. This observation corresponded with significantly less injury score in the cortical and medullary compartments of the kidney when compared to control group. The authors observed the same renal protection by siP53 in a rat model of cisplatin-induced acute kidney injury [137]. It is worth noting that proximal tubular epithelial cells are the first to undergo apoptosis, as they are the most sensitive to hypoxia and are also the primary site for siRNA uptake in the kidney. Therefore, p53 could be a therapeutic target in attenuating IRI in organ transplantation and other diseases. In a porcine model of kidney autotransplantation, Yang and colleagues [138] also showed that local and systemic administration of siRNA targeted at caspase-3 during 24 h of cold ischemia followed by reperfusion of renal autografts substantially downregulated caspase-3 mRNA and protein expression, reduced apoptosis and inflammation, and tubulointerstitial damage and improved renal function in comparison with two control groups that received either negative siRNA or no siRNA. In a rat model of lung transplantation, increased cold ischemic time positively correlated with apoptosis of graft cells after graft reperfusion via mitochondrial permeability transition pore opening along with protein kinase C (PKC) activation [21, 22]. Unsurprisingly, treatment with the PKC inhibitor, δV1-1, during prolonged cold ischemic time inhibited mitochondrial translocation of PKC and p53, and thus prevented apoptosis, while siRNA reduced cytokine production and further inhibited apoptosis [21]. These reports were later confirmed in human lung epithelial and endothelial cells in a simulated lung transplant setting [139] as well as in rat models of liver and lung transplantation in which diannexin (a recombinant human annexin V homodimer) was used [140, 141]. Following these promising experimental results, treatment with diannexin proved protective in a phase II clinical trial (NCT00615966) in kidney transplant recipients by reducing the incidence of DGF and days on dialysis compared to placebo-treated control group [142]. This has paved the way for testing of more apoptosis-based therapeutic agents in clinical organ transplantation. Along the same line of clinical investigation, plasma levels of the apoptotic markers, M30 and M65, were elevated at post-transplant days 1 and 2 in lung transplant recipients who developed grade 3 of PGD at 72 h, which were associated with increased duration of mechanical ventilation, longer hospital stay and higher mortality [143, 144]. Thus, these apoptotic markers may represent excellent diagnostic markers of PGD3 and a therapeutic target in organ transplantation.

Also, silencing of caspase-8 and Fas by inferior vena cava delivery of short hairpin RNA (shRNA) resulted in renal protection against IRI in a uninephrectomized mouse model in which renal artery was clamped for 60 min at 32 °C. Renal protection was characterized by reduced renal tubular injury score, and reduced levels of serum creatinine and blood urea nitrogen [145]. The deleterious role of caspase-3 and other pro-apoptotic factors was also observed in mouse model of DCD liver transplantation in which the authors observed severe liver allograft injury, which was hallmarked by increased Kupffer cell apoptosis, chemokine expression, and influx of inflammatory cells in liver allografts, high mortality of transplant recipient mice. This was associated with activation of TLR4-ERK1/2-Fas/FasL-caspase-3 signaling pathway in liver allografts [146]. However, infusion of mesenchymal stem cells significantly inhibited this pathway, reduced Kupffer cell apoptosis and inflammation, which ultimately culminated in prolonged recipient survival [146]. Using an in vitro model of hepatic IRI, primed human amnion-derived mesenchymal stromal/stem cells significantly inhibited the activation of caspase 3/7, and thus prevented apoptosis during early reperfusion period [147], which was later found to be enhanced by PPARβ/δ (peroxisome-proliferator-activated receptor) in myocardial IRI [148]. Another study also showed high expression of caspase-3-positive cells in liver allografts after reperfusion, which positively correlated with hypertransaminasemia, increased TNF-α level and mitochondrial ROS production, and contributed to allograft dysfunction [149], while IRI-induced apoptosis was also reported in acinar cells in experimental pancreas transplantation [150]. Collectively, these pieces of preclinical and clinical evidence suggest that targeting pro-apoptotic proteins in the apoptotic signaling pathways is a potential approach to attenuating or preventing IRI and complications in organ transplantation.

#### Autophagy in allograft ischemia–reperfusion injury

Autophagy is a regulated cellular process of self-degradation during which damaged cytoplasmic organelles or denatured proteins and other non-functional intracellular macromolecules are degraded by lysosomes through formation of double-membrane vesicles called autophagosomes or autophagic vacuoles, to which lysosomal enzymes are delivered. The products of lysosomal degradative action are reused for biosynthetic processes and energy production. Thus, autophagy is a homeostatic process which is mainly mediated by hormones and regulated by autophagy-related proteins, and could be considered as an inducible adaptive response to cellular stress such as hypoxia due to ischemia and oxidative stress during reperfusion after organ transplantation.

Autophagy plays an important role in protecting against IRI and functions to ensure cell survival during periods of starvation. However, dysregulation or excessive activation of autophagy, such as during prolonged cold preservation of organ grafts, triggers autophagic cell death without caspase involvement [151, 152]. In a rat model of orthotopic lung transplantation, preservation of lung allografts under cold ischemic condition for 1, 3, 6, 9, and 12 h led to increased ROS production, enhanced autophagy and aggravated lung IRI in a time-dependent manner via mammalian target of rapamycin (mTOR) signaling pathway [153]. However, addition of 3-methyladenine (3-MA; autophagy inhibitor) or oligomycin (ATP synthase inhibitor) to the lung preservation solution during cold ischemia decreased ROS production, autophagy and ameliorated lung IRI [154]. In the same study, supplementation of the lung preservation solution with rapamycin (autophagy activator) worsened lung IRI [154]. Reports from a mouse model of hepatic IRI demonstrated that short-term starvation induced expression of hepatocellular autophagy both in vivo and in vitro and ameliorated hepatic IRI via activation of Sirt1-autophagy signaling pathway. In accord, inhibition of Sirt1-autophagy pathway with sirtinol aggravated hepatic IRI [155]. In a rat model of hepatic IRI, intraperitoneal administration of baicalein (another autophagy activator) 1 h prior to warm ischemia improved liver function and preserved liver ultrastructure, with increased expression of the autophagic marker, LC3B-II, as well hepatic heme oxygenase (HO)-1 expression. Remarkably, inhibition of the baicalein-induced autophagy with 3-MA attenuated this protection [156]. Using primary rat hepatocytes, the same authors observed hepatocyte protection by baicalein-induced autophagy against hypoxia-reoxygenation injury, which was attenuated by 3-MA or siRNA targeted at Atg7 (autophagy effector enzyme). In addition, inhibition of HO-1 activity with tin protoporphyrin IX or HO-1 siRNA abolished baicalein-mediated autophagy and worsened hepatocellular injury [156]. It is important to note that at the ischemic phase of IRI when nutrient is depleted, autophagy degrades non-functional cytoplasmic proteins and other cellular structures, thereby promoting nutrient mobilization for biosynthesis and energy production (e.g. amino acids, nucleotides, lipids and carbohydrates) to support critical life activities of cells, and thus preventing apoptotic and necrotic cell death via activation of phosphoinositide 3-kinase/Akt/mTOR pathway [157, 158]. However, excessive activation of autophagy during reperfusion is unable to rescue cells when mitochondria generate ATP and Ca^2+^ accumulate in the mitochondria and autophagy is unable to completely neutralize reperfusion pressure, leading to cell damage and cell death [155].

#### Mitophagy in allograft ischemia–reperfusion injury

Considering the critical role of mitochondria in the regulation of cellular energy homeostasis and cell death as discussed in previous sections, damaged or dysfunctional mitochondria contribute significantly to oxidative stress and cell death through increased ROS production and release of pro-apoptotic proteins. Therefore, selective clearance of these organelles in a timely manner before they activate cell death is an essential process for cell survival and homeostasis. This selective removal of damaged or dysfunctional mitochondria is via autophagic mechanism termed *mitophagy*, and it serves as a mechanism to maintain mitochondrial quality and quantity control. Thus, mitophagy is a cytoprotective response that favors adaptation to stress such as ischemia and reperfusion. However, over-production of ROS and apoptotic proteases can inactivate mitophagy and cause cell death [159, 160]. Mitophagy is regulated by a signaling pathway known as PTEN-induced putative kinase 1 (PINK1)/Parkin pathway. PINK1 is a mitochondria-targeted kinase that protects cells from stress-induced mitochondrial dysfunction while Parkin is a cytosolic ubiquitin E3 ligase in the ubiquitin–proteasome system, tagging damaged and unwanted proteins with ubiquitin [161]. When mitochondria are damaged or become dysfunctional, mitochondrial translocation of PINK1 is limited to the mitochondrial outer membrane, where PINK1 recruits Parkin to mediate mitophagy via activation of its ubiquitin ligase activity, including recruitment of mitophagy receptors to link mitochondria to autophagosomes through interaction with LC3 protein for autophagic engulfment of the damaged or dysfunctional mitochondria and then degradation [161–163]. Several animal models of renal IRI and corresponding in vitro studies have shown the protective effects of PINK1 and Parkin and other inducers of mitophagy such as BNIP3. In these studies, knockout of PINK1, Parkin or BNIP3 genes abrogated mitophagy in renal proximal tubular epithelial cells and worsened renal IRI as evidenced by enhanced accumulation of damaged mitochondrial, ROS production, increased cell death and inflammatory response after IRI [164–166]. However, overexpression of these genes induced mitophagy, improved mitochondrial function, enhanced cell survival, and ultimately protected against renal IRI [164–166]. The upregulation of mitophagy by these genes and their protective effects were also observed in mouse models of cardiac and hepatic IRI [161, 167], human liver endothelial cells [167] and after ischemic preconditioning, where a brief period of non-lethal ischemia–reperfusion protected against subsequent prolonged period of IRI [168]. These laboratory findings suggest that mitophagy plays an important role in preserving mitochondrial quality control, cell survival and organ function during IRI, and therefore could represent an important protective mechanism against IRI and could be extended to organ transplantation setting by pharmacological interventions prior to or during ischemia and reperfusion of organ grafts.

## Graft Immunogenicity and immune response in organ transplantation

Cell death and IRI due to transplant procedure involving donor organ procurement, cold storage, and engraftment activate the innate immune system in which various components contribute to further cell death, graft inflammation, graft rejection, and ultimately negatively impacting both short- and long-term post-transplant outcomes. While apoptosis is non-inflammatory or protolerogenic and results in timely phagocytosis of dying cells without immune system activation [169], necrosis involves activation of the immune system in which damaged and dying cells release potent pro-inflammatory contents into the extracellular space before death [16–18]. In the transplanted graft, graft-infiltrating monocytes associated with the transplant procedure detect various stimuli that lead to a series of inflammatory events induced by danger molecules derived from dead allogeneic donor cells. As part of the mechanism of immunological defense, the innate immune system recognizes these danger molecules as pathogen-associated molecular patterns (PAMPs). The inflammatory response is also triggered by damage-associated molecular patterns (DAMPs) from the donor organ parenchyma after sterile inflammatory stimuli, which occurs during anastomosis in the organ transplant procedure [170, 171]. It has become evident that following the release of these danger molecules including ATP into the extracellular compartment, they induce immunometabolic changes in activated immune cells, causing these cells to redirect their metabolic flux since they have different bioenergetics and biosynthesis compared to resting cells [172–174]. As mentioned in the section on necroptosis, donor DAMPs (and PAMPs) are recognized by pattern recognition receptors (PRRs) such as Toll-like receptors (TLRs; e.g. TLR2 and TLR4) and complement receptors (another component of innate immunity) on the surface and cytoplasm of myeloid cells such as monocytes and macrophages, resulting in graft inflammation, development of acute and chronic graft rejection and early and long-term graft dysfunction [175–180]. Following recognition of PAMPs and DAMPs by PRRs, PRRs activate downstream signaling pathways, which in turn, activate innate immune responses and consequently initiate antigen-specific adaptive immune responses. This occurs due to the action of PRRs, producing pro-inflammatory cytokines and chemokines along with activation of costimulatory molecule expression and amplification of antigen-processing and antigen presentation by cells of the innate immune system [181–184]. Considering that activation of TLRs facilitate leukocyte migration and infiltration, as well as production of pro-inflammatory cytokines and chemokines and other pathological processes that result in graft rejection and dysfunction, research into the development of inhibitors that target TLR signaling pathway is gaining interest in the field of organ transplantation, and when successful, could open novel avenues to prevent or reduce post-transplant complications such as graft rejection, and improve both short- and long-term post-transplant outcomes.

## Conclusion

IRI is a critical pathological condition in which cell death plays a major contributory role, and negatively impacts post-transplant outcomes. As there are no therapeutic strategies currently available to prevent the development of IRI-induced allograft damage, inhibition of the various forms of cell death as discussed above, holds a great clinical promise in the quest to preventing IRI and improving allograft quality and function that would lead to a long-term success of organ transplantation. Also, some of the components in the mechanisms of cell death in IRI could represent non-invasive diagnostic markers for IRI-induced allograft complications such as delayed graft function, graft rejection, chronic graft rejection, primary graft dysfunction and chronic graft dysfunction.

## Data Availability

Not applicable.
